# Inflammation suppresses *DLG2* expression decreasing inflammasome formation

**DOI:** 10.1007/s00432-022-04029-7

**Published:** 2022-05-02

**Authors:** Simon Keane, Matthew Herring, Peter Rolny, Yvonne Wettergren, Katarina Ejeskär

**Affiliations:** 1 School of Health Science, DHEAR, Translational Medicine, University of Skövde, Skövde, Sweden; 2 Systems Biology Research Centre, School of Bioscience, University of Skövde, Skövde, Sweden; 3 Division of Gastroenterology/Hepatology, Department of Medicine, Sahlgrenska University Hospital/Östra, Gothenburg, Sweden; 4 Department of Surgery, The Sahlgrenska Academy at University of Gothenburg, SU/Östra, Gothenburg, Sweden

**Keywords:** DLG2, Inflammasome, NFKBIZ, Ulcerative colitis, Colon cancer

## Abstract

**Purpose:**

Loss of expression of *DLG2* has been identified in a number of cancers to contribute to the disease by resulting in increased tumor cell proliferation and poor survival. In light of the previous evidence that *DLG2* alters the cell cycle and affects proliferation, combined with indications that *DLG2* is involved in NLRP3 inflammasome axis we speculated that *DLG2* has an immune function. So far, there is no data that clearly elucidates this role, and this study was designed to investigate *DLG2* in inflammatory colon disease and in colon cancer as well as its impact on inflammasome induction.

**Methods:**

The *DLG2* expression levels were established in publicly available inflammation, colon cancer and mouse model datasets. The overexpression and silencing of *DLG2* in colon cancer cells were used to determine the effect of *DLG2* expression on the activation of the inflammasome and subsequent cytokine release.

**Results:**

The expression of *DLG2* is repressed in inflammatory colon diseases IBD and Ulcerative colitis as well as colorectal cancer tissue compared to healthy individuals. We subsequently show that induction with inflammatory agents in cell and animal models results in a biphasic alteration of *DLG2* with an initial increase followed by an ensuing decrease. *DLG2* overexpression leads to a significant increase in expression of IL1B, IκBζ and BAX, components that result in inflammasome formation. *DLG2* silencing in THP1 cells resulted in increased release of IL-6 into the microenvironment which once used to treat bystander COLO205 cells resulted in an increase in STAT3 phosphorylation and an increase proliferating cells and more cells in the G2/M phase. Restoration of *DLG2* to the colon resulted in reduced AKT and S6 signaling.

**Conclusion:**

*DLG2* expression is altered in response to inflammation in the gut as well as colon cancer, resulting in altered ability to form inflammasomes.

**Trial registration:**

NCT03072641.

**Supplementary Information:**

The online version contains supplementary material available at 10.1007/s00432-022-04029-7.

## Introduction

Colon cancer is one of the leading causes of cancer related deaths worldwide. In the western world, poor diets are increasing the incidences of obesity and altering the diversity of commensal bacteria (Singh et al. 2017). The increasing rates of obesity within global populations are increasing the total number of individuals at an elevated risk for developing colorectal cancer (Lund et al. 2011). Another factor leading to the increased incidences of colorectal cancer in the general population is advancing age (Kolligs 2016). Additionally, inflammatory bowel diseases such as Crohn’s disease and ulcerative colitis are also known factors that increase the risk of colorectal cancer development and subsequent mortality (Lund et al. 2011). It is important to note that there are generally considered to be two major pathways for development of colon cancer development, spontaneous (SCC) (Shi et al. 2020) also referred to as adenoma–carcinoma-sequence (Ozawa et al. 2021) and Colitis associated colon cancer (CAC) (Wang et al. 2020; Zhang et al. 2020a, b) referred to as inflammation–dysplasia–carcinoma-sequence (Ozawa et al. 2021). SCC results from otherwise healthy mucosa transforming into precancerous polyps, then progressing to adenocarcinomas and then cancerous lesions with a number of specific genetic alterations that occur at each transition step (Jones et al. 2008). One of the first alterations is the loss or mutation of APC followed by KRAS mutation and then loss of p53, with IL-27 loss compounding the effect of p53 loss in other cancer forms (Dibra et al. 2016a, b). With CAC the order of alterations differs, resulting in flatter lesions with varying degrees of dysplasia. In CAC the loss of p53 occurs earlier in this process and APC later (Jones et al. 2008). Common to both pathways is the activation of COX-2 in response to inflammation (Sharma et al. 2001; Chu et al. 2004; Janakiram and Rao 2009). Underpinning the transitions is a constitutive activation of NFκβ signaling which in this context results in tumor cell survival, proliferation and cell cycle progression (Curtin et al. 2010; Shi et al. 2019).

The colon is home to the largest population of microbes in the human body and encounters the highest concentration of pathogens, representing a large risk of infection if there is a disturbance in the microbiota (Jahani-Sherafat et al. 2018). Therefore, the colon has a large number of immune cells present. The innate immune system is triggered through pattern recognition receptors (PRRs), which include Toll like receptors (TLR) and retinoic acid inducible gene-I (RIG-I) (Ostvik et al. 2020). PRRs, TLRs and RIGs activate the inflammatory pathways in response to Pathogen Associated Molecular Patterns (PAMPs) (Santiago et al. 2020) or Damage Associated Molecular Patterns (DAMPs) (Matzinger 1994; Zhang et al. 2010). The upregulation of NFκβ in response to a DAMP or PAMP initiates the upregulation of proinflammatory factors required for priming of the *NLRP3* inflammasome, by inducing the upregulation of IL-1β (Bank et al. 2014; Hai Ping et al. 2016). The activation of the inflammasome requires a second signal such as ATP (Jang et al. 2021). Mutations in the components of the inflammasomes often result in an increased susceptibility to cancer. The *Q705K* SNP in *NLRP3* is associated with higher inflammasome activation (Verma et al. 2012) and poor patient survival in advanced stage colorectal cancer (Ungerback et al. 2012). It has been proposed that this SNP combined with external stimuli can result in increased IL-1β and IL-18 production (Verma et al. 2012), and IL-1β has been shown to downregulate pTEN by activation of NFκβ in colon cancer (Hai Ping et al. 2016), whilst IL-18 contributes to inflammasome mediated protection against tumorigenesis in colitis patients (Zaki et al. 2010). IL-18 has also been shown to be activated downstream of the retinoic acid receptor (RAR) as well as promote T- Helper 17 (Th 17) responses. The role of *NLRP3* in colon cancer has been controversial with studies showing that high expression of *NLRP3* drives epithelial-mesenchymal transition (EMT) (Shao et al. 2020) and results in poor survival (Shi et al. 2021). However, in direct contradiction another study has shown that NLRP3 mediates inhibition of metastatic growth (Dupaul-Chicoine et al. 2015). It is however important to note that the expression of NLRP3 and activation of the inflammasome differs with the type of cell mediating the inflammasome effect (Jang et al. 2021). Previous studies have shown that inflammasome activation requires *NFKBIZ* (IκBζ)*,* a lesser known nuclear Factor kappa B (NFκβ) inhibitor, that regulates transcription of NFκβ targets by binding p50 or p52 subunits of NFκβ (Yamazaki et al. 2001). Furthermore, it can directly bind and negatively regulate STAT3 and induce apoptosis (Willems et al. 2016). Loss or altered *NFKBIZ* results in chronic inflammation by inhibiting the production of IL-10 (Horber et al. 2016) as well as diminished inflammasome priming (Kim et al. 2020). Chronic and/or overactivation of the inflammasome and resulting increase in IL-6 expression result in poor outcomes in colon cancer patients by activating STAT3 (Corvinus et al. 2005; Slattery et al. 2007; Xiong et al. 2008). When taken together this highlights the dual nature and the importance of concise control of the inflammasome and inflammatory responses in human colorectal cancers.

Recently, low *DLG2* expression in osteosarcoma (Shao et al. 2019), ovarian cancer (Zhuang et al. 2019) and neuroblastoma (Keane et al. 2020, 2021; Siaw et al. 2020) has been identified as contributing to disease etiology, with low *DLG2* expression in neuroblastoma showing increased cell proliferation and poor survival (Keane et al. 2020). Additionally, preliminary data show that *DLG2* induces cell death by modulating BAX/BCL2 in response to DNA damage (Keane et al. 2022), colocalizing in the same pathways as the NLRP3 inflammasome. It has been shown that *DLG2* can be induced by treatment with 13´cis retinoic acid (Siaw et al. 2020) with retinoic acid response element 1 (*RARRES1)* directly regulating *DLG2* (Sahab et al. 2010). Additionally, DLG2 induces p53 mediated cell death in response to UVC irradiation (Keane et al. 2022).

In light of the building body of evidence that *DLG2* is an important tumor suppressor gene we here investigate *DLG2* and *NFKBIZ* and their impact on the inflammasome induction in inflammatory colon disease and in colon cancer. We detail the importance of *DLG2* in activating the inflammasome complex and the effect of inflammation on *DLG2*.

## Methods

### Gene expression analysis

Data for analyses and comparison of *DLG2* expression between the different patient subgroups was imported from the R2 platform (http://r2.amc.nl). The six independent colon cohorts; (Galamb et al. 2008) (GSE4183), (Haberman et al. 2019) (GSE109142), (Vancamelbeke et al. 2017) (GSE75214), (Jiang et al. 2008) (GSE10950), (Sabates-Beliver et al. 2007) (GSE8671) and (Agesen et al. 2012) (GSE24551). The inflammation time series mouse models, DSS (Fang et al. 2012) (GSE22307) and Colon T cell transfer (Fang et al. 2011) (GSE27302). The microarray data was downloaded as the centered log2 fold change.

### Cell Lines and cell culture

Human colon adenocarcinoma cell lines SW480 and COLO205 were obtained from ATCC Cell Line Collection and THP 1-ASC-GFP monocytes were obtained from Invovogen. The SW480 and COLO205 cell lines were maintained in RPMI 1640 (ThermoFisher Scientific) supplemented with 10% FBS, 1% L-Glutamine (ThermoFisher Scientific), 10 mM HEPES solution (ThermoFisher Scientific) and 1 mM sodium pyruvate (ThermoFisher Scientific). THP 1-ASC-GFP cells were cultured in RPMI-1640 with L-glutamine (Merck), 10% heat inactivated premium grade FBS (Biowest), 10 mM HEPES, 1 mM sodium pyruvate (Merck), 0,45% glucose (Merck) and 100U/ml penicillin–streptomycin (Merck) at 37 °C and 5% CO_2_. Zeocin (200 µg/ml) (Invivogen) was added to the culture medium as per the manufacturer’s instructions. Cell density was maintained between 5 × 10^5^ and 1.5 × 10^6^ cells/ml and cells were used up to passage number ten. Differentiation was conducted with 100 ng/ml PMA (Merck) for 72 h followed by 72 h of rest in fresh media. Priming of undifferentiated or differentiated cells was conducted with 500 ng/ml ultrapure LPS (Invivogen) for up to 24 h as indicated. Activation of inflammasome complex formation was conducted with 5 mM ATP (Merck) for 30 min.

### Plasmids, siRNAs and transfections

*DLG2* (NM_001351274.2) overexpression plasmids on a backbone of pCMV6-AC-GFP (catalogue # PS100010) vector were purchased from Origene Technologies. siRNA targeting *DLG2* (s4122) or Silencer™ Select Negative control No. 1 siRNA (4,390,843) was purchased from Ambion (ThermoFischer Scientific). SW480 cells were grown to 80% confluence and subsequently transfected with; *DLG2* plasmid, empty vector “mock” (pCMV6-AC-GFP), si-*DLG2* or scrambled control “mock”. 100 ng plasmid-DNA or 10 pmol siRNA was complexed with 0.3 µl of Lipofectamine 2000 according to the Lipofectamine 2000 reagent forward transfection protocol (Invitrogen; ThermoFisher Scientific).

### Inclusion of study subjects

Study subjects who underwent colonoscopy at the Sahlgrenska University Hospital, Gothenburg, Sweden were consecutively included in the study. Reasons for referral to colonoscopy for each participant are presented in Additional file 1. Forty controls and twenty patients who were diagnosed with colon cancer were included. The prerequisite for inclusion into the control group was ≥ 18 years of age, and a normal-appearing mucosa in the entire colon, e.g. patients with any significant pathology such as colonic polyps or adenomas, inflammatory bowel disease, malignancy, ischemic colitis etc. were excluded. Possibility of microscopic colitis was ruled out by light microscopic examination of biopsy specimens obtained from the mid-portion of the ascending colon as well as from the sigmoid. Presence of colonic diverticula was accepted provided there were no signs of acute diverticulitis and/or diverticulosis-associated colitis. The prerequisite for inclusion into the colon cancer group was the presence of at least one malignant tumor in the colon and ≥ 18 years of age. Tumors were classified according to the Tumor–Node–Metastasis (TNM) staging system (Compton et al. 2000).

### Collection of tissue samples

During the diagnosis colonoscopy, mucosa samples were obtained from the mid-portion of the ascending colon (right side samples) as well from the sigmoid (left side samples) using a regular biopsy forceps. If applicable, a tissue sample was also collected from the tumor. The distance between the tumor and the matching cancer mucosa that was sampled on the same side as the tumor was approximately 10 cm. Tissue samples were frozen immediately in liquid nitrogen, and stored at − 80 °C until used.

### Bacterial treatment in flies

The strain white (w-1118) (Bloomington Drosophila Stock Center) were used in the study. Newly laid eggs (within 3 h) were transferred to apple-agar plates with either control food (15% dry yeast, 17% mashed potato powder, 14% apple juice, 3% sugar, 1 grinded Probion placebo tablet per 10 g of food) or food supplemented with Probion Clinica (15% dry yeast, 17% mashed potato powder, 14% apple juice, 3% sugar, 1 grinded Probion Clinica tablet per 10 g of food). Larvae raised on control food were transferred to food supplemented with Probion Clinica after 1, 2, 3 or 4 days. After 5 days the larvae gut from 5 five larvae from each experiment were dissected and used for RNA extraction.

One Probion Clinica (Wasa Medicals AB, Halmstad, Sweden) tablet includes 7 × 10^9^ CFUs *Bifidobacterium lactis* Bl-04 (ATCC SD5219), 3,5 × 10^9^ CFUs *Lactobacillus acidophilus* NCFM (ATCC 700396) and 0.32 g inulin/xanthan mix.

### DNA and RNA isolation

RNA was isolated from tissue samples using Qiagen AllPrep DNA/RNA/Protein Kit according to the manufacturer’s instructions. The samples were kept at – 20 °C until analysis. cDNA was synthesized from total RNA using the High Capacity cDNA Reverse Transcription Kit (no. 4368814, ThermoFisher Scientific) and run on a Bio-Rad T100 Thermal Cycler (Bio-Rad laboratories). RNA from SW480 and THP-1 cells were extracted with RNeasy Kit (Qiagen) according to manufacturer’s protocol. RNA was quantified by NanoDrop (NanoDrop Technologies) and 2 µg of RNA was reverse-transcribed into double stranded cDNA on a T-professional Basic Gradient thermal cycler (Biometra) using the High Capacity cDNA Reverse Transcription kit (Applied Biosystems). cDNA corresponding to 20 ng of RNA was used for each qPCR reaction.

### Quantitative PCR analysis

The relative gene expression of selected genes was quantified using TaqMan^®^ Assays labelled with FAM-MGB (ThermoFisher Scientific) (Table 1) or by SYBR green (Table 2). Samples were run as duplicates in 96-well plates. Polymerase chain reactions were carried out in 5 μl reactions with 1 × TaqMan™ Gene Expression Master Mix (no. 4369016, ThermoFisher Scientific), 1 × gene-specific assay and 2.5 μl cDNA. The plates were run and analyzed using the Pikoreal qPCR System (ThermoFisher Scientific) according to the manufacturer’s protocol. Thresholds and baselines were set manually and Ct values were extracted. All Ct values were normalized to the mean of the reference genes*; ACTb, GAPDH, GUSB* and *PPIA* (Δ*C*_t_) for each sample.

**Table 1:** List of TaqMan® assays

Gene name	Product number
PPIA	Hs99999904_m1
GUSB	Hs00939627_m1
GAPDH	Hs02758991_m1
ACTb	Hs99999903_m1
NFKBIZ	Hs00230071_m1
RELA	Hs00153294_m1
RELB	Hs00232399_m1
DLG2	Hs00265843_m1
IL-1B	Hs01555410_m1
IL-6	Hs00174131_m1
BCL2	Hs00608023_m1
BAX	Hs00180269_m1
FOXO3	Hs00818121_m1
STAT3	Hs00374280_m1

**Table 2:** SYBR primer pairs

IL-1b	Forward	ATGATGGCTTATTACAGTGGCAA	PrimerBank ID 27894305c1
IL-1b	Reverse	GTCGGAGATTCGTAGCTGGA	PrimerBank ID 27894305c1
NLRP3	Forward	GATCTTCGCTGCGATCAACAG	PrimerBank ID 208879435c1
NLRP3	Reverse	CGTGCATTATCTGAACCCCAC	PrimerBank ID 208879435c1
NFkB1	Forward	AACAGAGAGGATTTCGTTTCCG	PrimerBank—259155300c1
NFkB1	Reverse	TTTGACCTGAGGGTAAGACTTCT	PrimerBank—259155300c1

### Protein analysis by Western blot

Protein was extracted from the transfected cells in 96 well plates (1 × 10^4^ cells/well), by aspirating the media and incubating on ice for 5 min then adding ice cold RIPA buffer (ThermoFisher Scientific, 89901). Western blot analysis was performed using a Mini-PROTEAN^®^ TGX™ 8–20% gradient gel (Bio-Rad), protein was blotted onto LF-PVDF membrane (8 min, 25 V and 2.5A) using a Trans-Blot^®^ Turbo™ Transfer System (Bio-Rad). Blots were subsequently blocked for 1 h in 5% milk in TBST buffer (0.1% Tween-20 and 150 mM NaCl in 10 mM Tris–HCl, pH 7.4) as per the manufacturer’s recommendations. Primary antibodies (Table 3) were diluted in PBST (0.1% Tween-20 in PBS). After incubation with the primary antibodies the membranes were washed 3 × 10 min in TBST 0.1% (0.1% Tween-20 in tris-buffered saline). Secondary antibodies; Starbright B520 goat anti-rabbit (12,005,870, 1:5000, BioRad) and Starbright B700 goat anti-mouse (12,004,159, 1:5000, BioRad) were incubated for 1 h at RT. Transient transfection and Western blot analyses were performed in triplicate as three independent experiments. Image detection was performed on ChemiDoc MP (BioRad) and band intensity was quantified using Image lab™ (v. 6.1, BioRad). Protein loading from the different experiments and gels were normalized against total loaded protein from stain free images. The p-STAT, p-AKT, p-FOXO3 and p-S6 levels were calculated relative to the total STAT (STAT), total AKT (AKT), total FOXO3 (FOXO3) and total S6 (S6) protein quantities respectively. GAPDH was included as loading control.

**Table 3:** Primary antibodies with weights, dilutions and manufacturer

Gene	Product number	Weight (kDa)	Dilution	Manufacturer
DLG2	19046S	115	1:500	CST
p-Akt1/2/3 (Ser 473)	sc-514032	52	1:500	Santa Cruz Biotechnology
AKT-1	sc-5298	52	1:1000	Santa Cruz Biotechnology
p-S6 (Ser235/236)	4858S	32	1:1000	CST
S6	2317S	32	1:1000	CST
p-STAT3 (Ser727)	44384G	90	1:1000	Invitrogen
STAT3	MA1-13,042	90	1:3000	Invitrogen
p-FOXO3a (Ser 318/321)	9465S	80	1:1000	CST
FOXO3a	99199S	80	1:1000	CST
IL-6	CPTC-il6-1-s	24	0.5 µg/ml	DSHB
IL-1β	sc-12742	23	1:200	Santa Cruz Biotechnology
TIG1	sc-390461	33	1:100	Santa Cruz Biotechnology
BCL2	sc-509	27	1:500	Santa Cruz Biotechnology
BAX	sc-20067	20	1:1000	Santa Cruz Biotechnology
p-P65 (Ser 536)	sc-136548	65	1:100	Santa Cruz Biotechnology
RELA	PCRP-RELA-2B6-s	65	0.5 µg/ml	DSHB
RELB	sc-48366	70	1:200	Santa Cruz Biotechnology
p-P105 (Ser 932)	sc-293141	85	1:500	Santa Cruz Biotechnology
NFKB1	sc-8414	50	1:200	Santa Cruz Biotechnology
NFKBIZ	9244	85	1:1000	Santa Cruz Biotechnology
GAPDH	12,004,168	37	1:2500	BioRad

### Quantification of inflammasome formation by microscopy

5 µM DRAQ5™ nuclear stain (ThermoFisher Scientific) was added to the cells 5 min before imaging. Live cell imaging was performed at 37 °C, 5% CO_2_ using the Olympus scanR High-Content Screening Station and CellR software, UPLSAPO 20 × objective and Hamamatsu C8484 CCD camera. FITC and Cy5 fluorophore channels were used to visualize PYCARD-GFP and DRAQ5™ nuclear stain respectively. Each stimulation condition was imaged at 72 locations. Replicate experiments were performed on fresh aliquots of cells cultivated, treated and imaged at separate times.

### Image analysis (FIJI)

Inflammasomes were identified using WEKA Trainable Segmentation plugin. The plugin was trained using 30 images of varying conditions and verified under all conditions. Identified specks were counted using the particle analyzer plugin. Cell nuclei were counted and used to determine total number of cells. Briefly, Gaussian blur (sigma = 2) was applied to images followed by an auto threshold step. Images were then converted to a binary image followed by 2-D watershed and the particle analyzer plugin was used to count cell nuclei.

### Statistical analysis

All data presented are plotted as Tukey’s box and whisker plots showing IQR, line at the median, + at the mean with whiskers ± 1.5-fold of interquartile range from at least three independent experiments, or as a Kaplan–Meier to determine event free survival over time. For all multi-group analyses, differences were determined by one-way ANOVA test followed by Holm–Sidak’s multiple comparison test. For comparisons between two groups a Mann–Whitney *U* test was used: *p < 0.05, **p < 0.01, ***p < 0.001. All analyses were conducted using GraphPad Prism version 8.0.1 for Windows, (GraphPad Software, http://www.graphpad.com).

## Results

### *DLG2* expression was low in inflamed tissues and in colorectal tumors, whereas *NLRP3* and *NFKBIZ* expressions were high in inflamed tissues

**Fig. 1:**
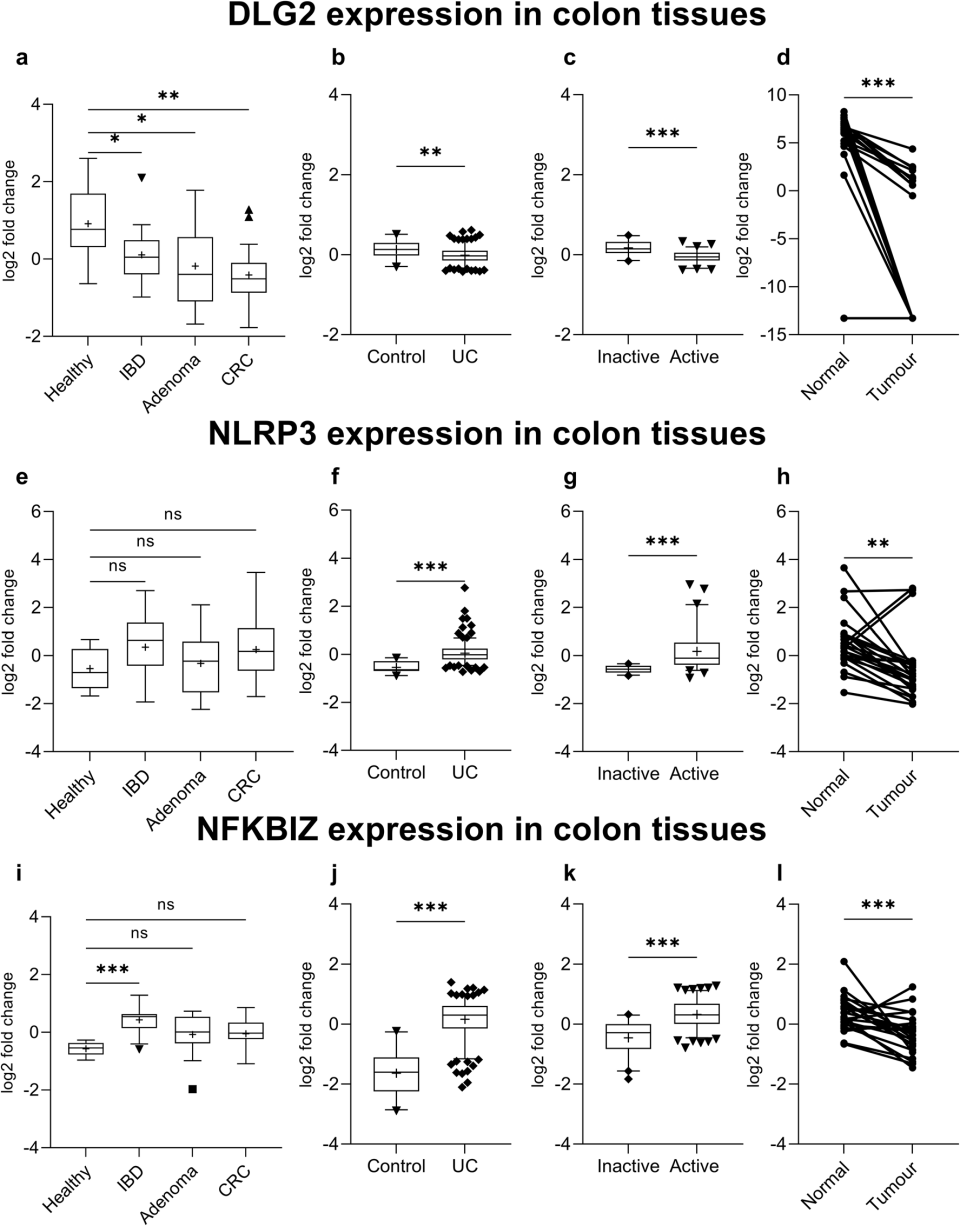
The alteration of *DLG2*, *NLRP3* and *NFKBIZ* gene expression in colon inflammation and cancer. **a** *DLG2*, **e** *NLRP3* and **i** *NFKBIZ* gene expression by sample type including; 8 Healthy patients, 15 IBD patients, 15 adenoma patients and 15 colon cancer patients (cohort GSE4183). **b** *DLG2*, **f** *NLRP3*, and **j** *NFKBIZ* expression in 20 healthy individuals compared to 206 with Ulcerative colitis (UC) (cohort GSE109142). **c** *DLG2*, **g** *NLRP3* and **k** *NFKBIZ* expression in 73 patients UC patients with active disease compared to 23 patients with inactive disease (cohort GSE75214). **d** *DLG2*, **h** *NLRP3* and **l** *NFKBIZ* expression of 24 case-controlled tumor mucosa samples (cohort GSE10950). The expression data are presented as median centered log2 fold change and plotted as Tukeys box and whisker plots showing IQR, line at the median, + at the mean with whiskers ± 1.5-fold of interquartile range. Data outside the whiskers are shown as outliers. The paired data are shown as an individual symbol with a connecting line. *p < 0.05, **p < 0.01, ***p < 0.001

We evaluated the expression of *DLG2*, *NLRP3* and *NFKBIZ* genes using publicly available microarray data for the independent colon cohorts (GSE4183; Fig. 1a, e, i) (Galamb et al. 2008), (GSE109142; Fig. 1b, f, j) (Haberman et al. 2019), (GSE75214; Fig. 1c, g, k) (Vancamelbeke et al. 2017), (GSE10950; Fig. 1d, h, l) (Jiang et al. 2008), obtained from the R2 Genomics Analysis and Visualization Platform (http://r2.amc.nl). In the different datasets gene expression was investigated in samples from patients with inflammatory bowel disease (IBD), adenoma or colon cancer compared to healthy controls (Fig. 1a); ulcerative colitis (UC) patients compared to controls (Fig. 1b); or ulcerative colitis (UC) patients with active vs. inactive disease state (Fig. 1c) and case controlled colorectal tumor samples compared to paired healthy mucosa (Fig. 1d). *DLG2* was downregulated in IBD (log2 FC = 0.81, *p* < 0.05), adenoma (log2 FC = 1.1, *p* < 0.1) and colon cancer (log2 FC = 1.3, *p* < 0.01; Fig. 1a). *DLG2* also showed a decrease in expression in UC compared to the control (log2 FC = 0.14, *p* < 0.01; Fig. 1b) and further decrease in expression when UC was active (log2 FC = 0.22, *p* < 0.001; Fig. 1c). A large downregulation in *DLG2* was seen in the paired healthy-tumor colon tissue from colorectal cancer patients (log2 FC = 12.6, *p* < 0.001; Fig. 1d). There was no difference in *NLRP3* expression in samples from patients with IBD, adenoma or colon cancer compared to healthy controls (Fig. 1e)*.* An increased *NLRP3* expression was detected in UC compared to control samples (log2 FC = 0.59, *p* < 0.001; Fig. 1f), and a further increase in *NLRP3* expression when the UC was active (log2 FC = 0.74, *p* < 0.001; Fig. 1g). A lower *NLRP3* expression was seen in the colon tissue compared to paired healthy mucosa in colorectal cancer patients (log2 FC = 1.1, *p* < 0.01; Fig. 1h). The *NFKBIZ* expression was higher in IBD samples compared to controls (log2 FC = 0.59, *p* < 0.001; Fig. 1i), however, no difference from controls was detected in adenoma or colon cancer samples (Fig. 1i). There was also an increased *NFKBIZ* expression in UC samples compared to the control (log2 FC = 1.8, *p* < 0.001; Fig. 1j) and a further increase in *NFKBIZ* expression when UC was active (log2 FC = 0.78, *p* < 0.001; Fig. 1k). However, a downregulation in *NFKBIZ* was seen in the paired tumor tissue compared to healthy mucosa from colorectal cancer patients (log2 FC = 0.52, *p* < 0.001; Fig. 1l). Additional cytokines were investigated with *IL1RN* showing increased expression in IBD, adenoma and CRC compared to healthy controls, however no significant difference between groups in *TGFB1*, *IL4*, *IL10*, *IL13*, *IL27* or *IL37* expression (Supplementary Fig. 1).

### *DLG2* expression was initially upregulated followed by downregulation over time in response to inflammatory signals

**Fig. 2:**
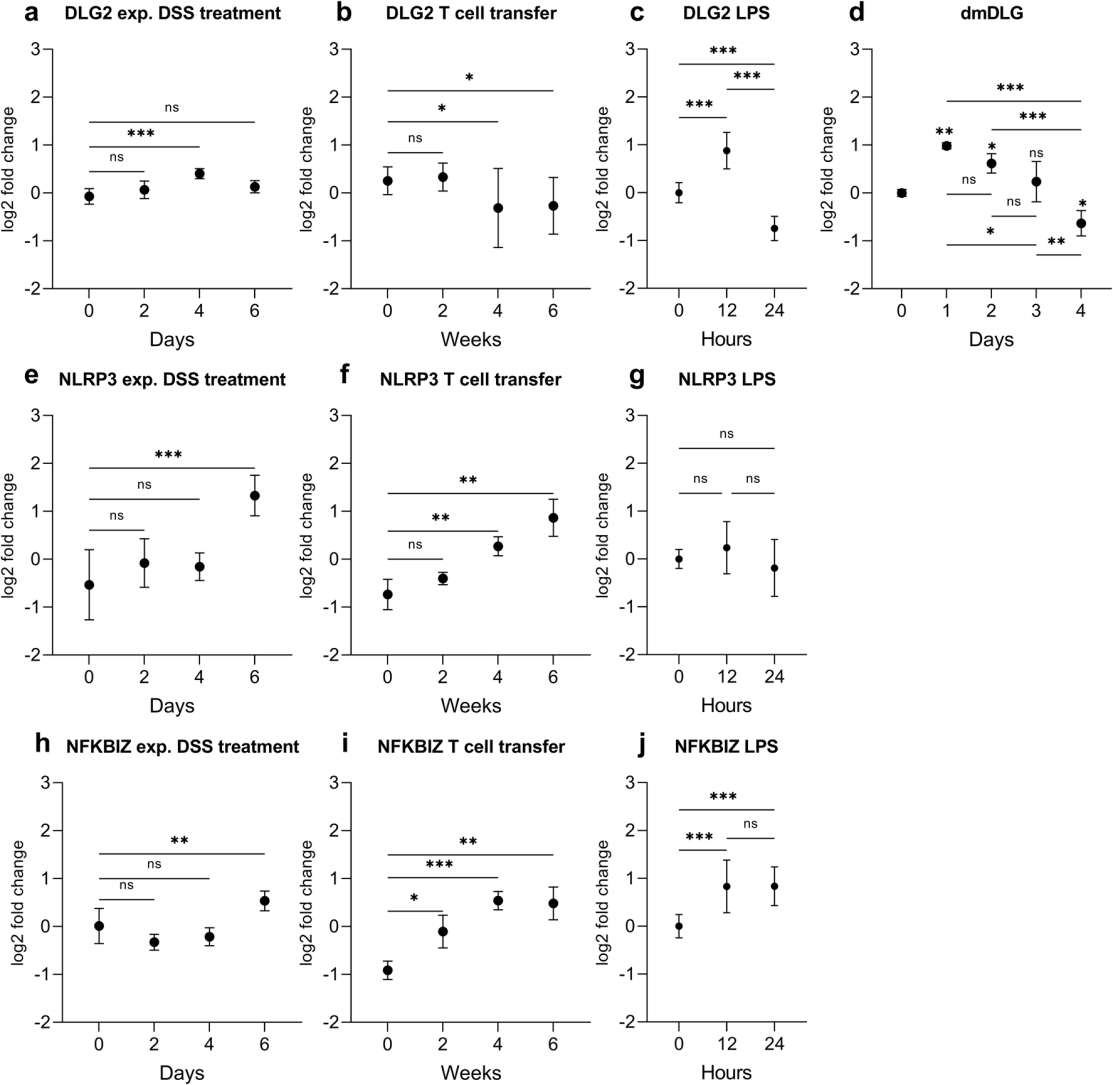
The response of *DLG2*, *NLRP3* and *NFKBIZ* gene expression to inflammation in mouse, cell and fly models. **a** *DLG2*, **e** *NLRP3* and **h** *NFKBIZ* gene expression in the colon tissue of 5 mice for each time point in response to DSS treatment at 0, 2-, 4- and 6-days post treatment (cohort GSE22307). **b** *DLG2*, **f** *NLRP3*, and **i** *NFKBIZ* expression in the colon tissue of 4 mice in response to T-cell transfer at 0, 2-, 4- and 6- weeks post transfer (cohort GSE27302). **c** *DLG2*, **g** *NLRP3* and **j** *NFKBIZ* expression in THP1 cells in response to LPS treatment at 0, 12 and 24 h. **d** The expression of dmDLG in *Drosophila melanogaster* gut in response to lactate bacteria treatment at 0, 1-, 2-, 3-, 4- days post treatment. The expression data are presented as median centered log2 fold change and plotted as mean ± SD. *p < 0.05, **p < 0.01, ***p < 0.001

We evaluated the expression of *DLG2*, *NLRP3* and *NFKBIZ* genes using publicly available microarray data in mouse colon from mice treated with Dextran Sulfate Sodium (DSS) to induce a colitis like phenotype (Fang et al. 2012) (GSE22307) and T cell transfer (Fang et al. 2011) (GSE27302) to model chronic colitis, obtained from the R2 Genomics Analysis and Visualization Platform (http://r2.amc.nl). *DLG2* was upregulated in the colitis mouse model 4 days after DSS treatment with no difference between 0 and 6 days of DSS treatment (log2 FC = 0.47, *p* < 0.001) (Fig. 2a). When given a T-cell transfer, *DLG2* expression in mice was decreased after 4 and 6 weeks (log2 FC = 0.57, *p* < 0.05 and log2 FC = 0.52, *p* < 0.05, respectively; Fig. 2b). When THP-1 monocytes were treated with Lipopolysaccharides (LPS) to induce immune responses, there was an initial increase in *DLG2* expression 12 h after exposure (log2 FC = 0.879, *p* < 0.001; Fig. 2c) then a decrease in *DLG2* was detected 24 h post exposure (log2 FC = 1.63, *p* < 0.001; Fig. 2c). The expression of the *Drosophila melanogaster* DLG2 ortholog dmDLG increased in fly larvae gut cells in response to *Bifidobacterium lactis* Bl-04 and *Lactobacillus acidophilus* NCFM, 24 h post treatment (log2 FC = 0.98, *p* < 0.01; Fig. 2d), with a progressive and gradual decrease in dmDLG over time until four days post treatment (log2 FC = − 0.64, *p* < 0.05; Fig. 2d). *NLRP3* expression increased after six days of DSS treatment (log2 FC = 1.9, *p* < 0.001; Fig. 2e) and after T cell transfer by increasing expression between 4 and 6 weeks after treatment (log2 FC = 1.0, *p* < 0.01 and log2 FC = 1.6, *p* < 0.01, respectively; Fig. 2f). When THP-1 cells were treated with LPS there was no alteration in *NLRP3* expression over time (Fig. 2g). *NFKBIZ* expression responded to DSS treatment after six days (log2 FC = 0.52, *p* < 0.01; Fig. 2h) and to T cell transfer by increasing its expression across all time points against the control up to 6 weeks after treatment (log2 FC = 0.80, *p* < 0.05, log2 FC = 1.5, *p* < 0.001 and log2 FC = 1.4, *p* < 0.01, respectively; Fig. 2i). When THP-1 cells were treated with LPS there was an initial increase in *NFKBIZ* expression 12 h after exposure (log2 FC = 0.83, *p* < 0.001; Fig. 2j) with the increase sustained 24 h post exposure (log2 FC = 0.84, *p* < 0.001; Fig. 2j).

### DLG2 overexpression results in increased NFKB components

**Fig. 3:**
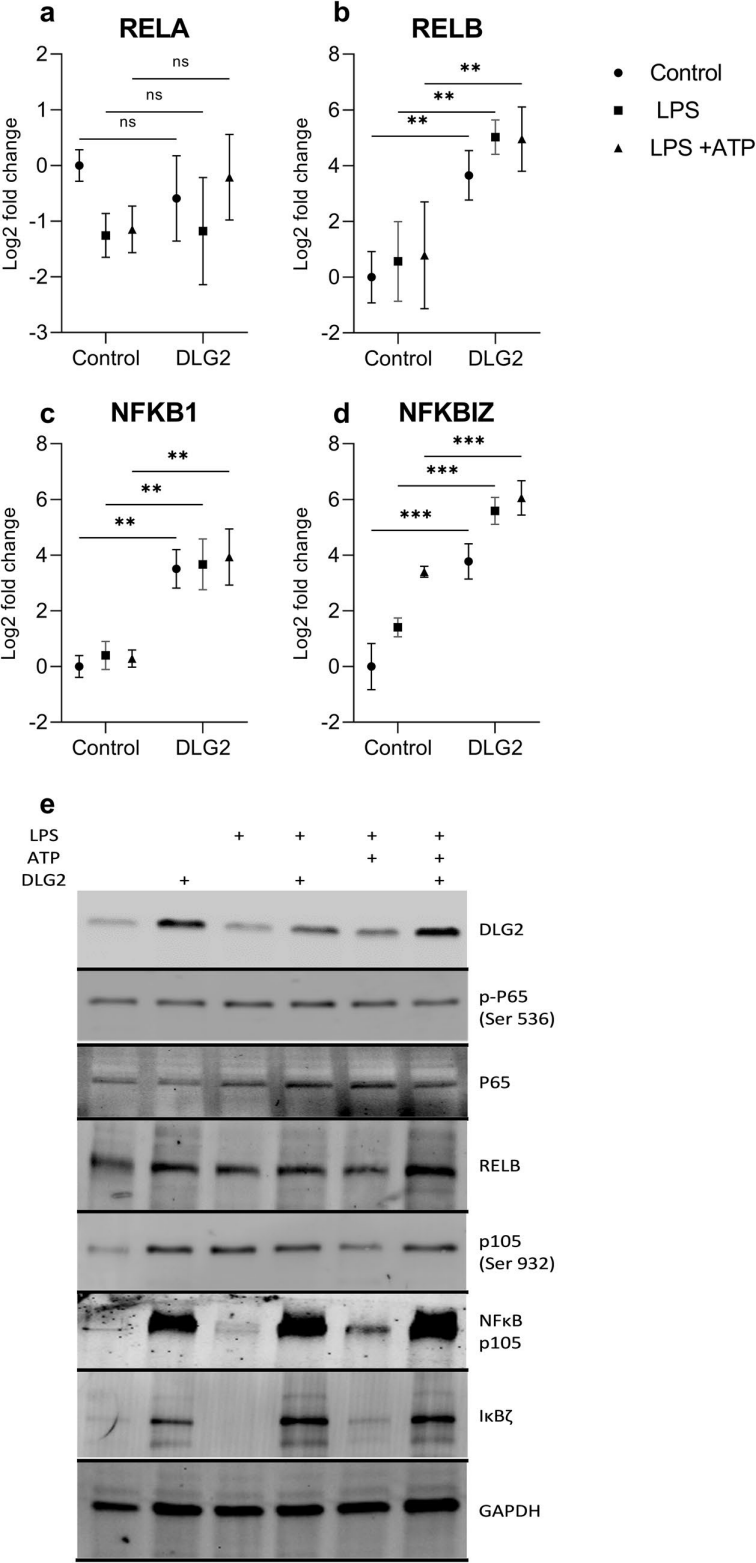
The response of NFκβ members to increased *DLG2* expression and activation of the inflammasome in THP-1 cells. The expression of **a** *RELA*, **b** *RELB*, **c** *NFKB1* and **d** *NFKBIZ* in response to control conditions (circle symbol), LPS priming (square symbol) and LPS + ATP treatment (triangle symbol) with or without *DLG2* overexpression. **e** Representative immunoblot showing the expression of; DLG2, p-P65 (Ser 536), RELA, RELB, p-P105 (Ser 932), NFκB1, IκBζ and GAPDH. Each experiment was performed in triplicate. The gene expression data are presented as log2 fold change and plotted as mean ± SD. *p < 0.05, **p < 0.01, ***p < 0.001

Using differentiated THP-1 monocytes we compared mock transfection to *DLG2* overexpressed and subsequent activation with either growth media, LPS or LPS with ATP. Gene expression analysis of *NFKB1*, *NFKBIZ*, *RELA* and *RELB* (Fig. 3 a-d) was performed in response to the growth conditions. We determined that there was no difference in *RELA* expression between the control and the *DLG2* transfected cells (Fig. 3a). *RELB* showed a consistent upregulation in response to *DLG2* overexpression, a stronger effect than both LPS and ATP treatments had (log2FC = 3.55, *p* < 0.01, log2FC = 4.46, *p* < 0.01 and log2FC = 4.17, *p* < 0.01; Fig. 3b). We investigated the expression of *NFKB1* and showed that like *RELB*, the expression was consistently upregulated in the *DLG2* expressed cells, with no additional effect by addition of LPS or ATP (log2FC = 3.51, *p* < 0.01, log2FC = 3.27, *p* < 0.01 and log2FC = 3.65, *p* < 0.01; Fig. 3c). Finally, we investigated the expression of *NFKBIZ* which was upregulated across all of the activations compared to the control with *DLG2* overexpressed cells showing higher expression (log2FC = 3.77, *p* < 0.001, log2FC = 4.18, *p* < 0.001 and log2FC = 2.667, *p* < 0.001; Fig. 3d). We subsequently confirmed that the effects of *DLG2* overexpression seen on gene expression level, also affected the protein expression, visualized by immunoblot for; DLG2, p-P65 Ser536, RELA, RELB, p-P105 Ser932, NFκB (P105), and IκBζ, using GAPDH as loading control (Fig. 3e). With the p-P65 Ser536 immunoblot indicating a low level of P65-phosphorylation across all activations. Despite the p-P105 Ser932 immunoblot detected higher amounts of phosphorylated NFκB (P105) in the DLG2 transfected cells, these transfections maintained lower relative phosphorylation compared to total amount of NFκB (P105) (Fig. 3e). DLG2 stimulated inflammasome formation and increased apoptosis in macrophage like cells.

### DLG2 overexpression results in inflammasome formation in THP1 cells

**Fig. 4:**
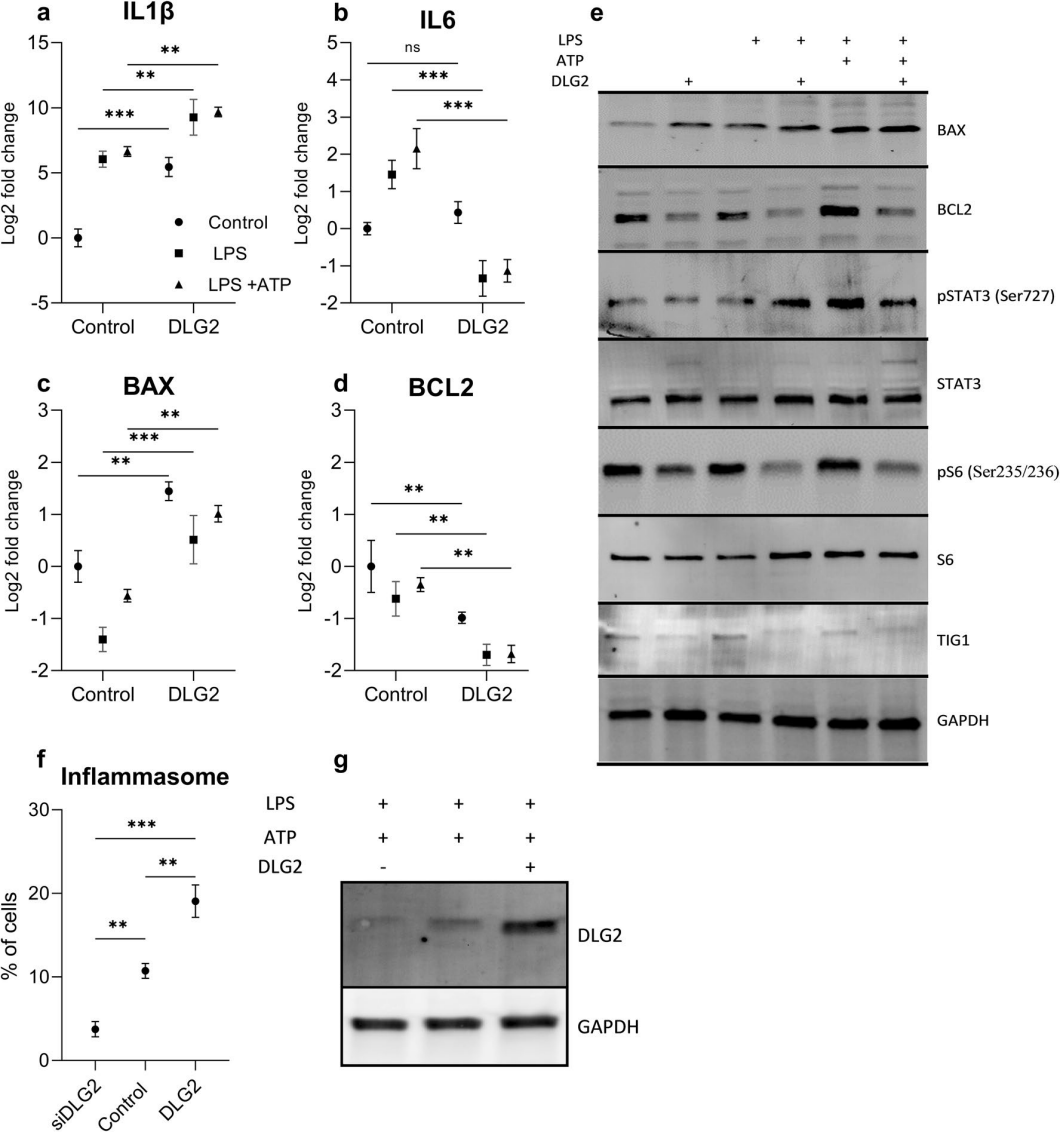
The response of cytokines and apoptotic proteins to increased DLG2 level and formation of the inflammasome in THP-1 cells. The gene expression of **a** *IL1B*, **b** *IL6*, **c** *BAX* and **d** *BCL2* in response to control conditions (circle symbol), LPS priming (square symbol) and LPS + ATP treatment (triangle symbol) with or without *DLG2* overexpression. **e** Representative immunoblot showing the expression of; BAX, BCL2, p-STAT3, STAT3, p-S6, S6, TIG1 and GAPDH. **f** Activation of the inflammasome in *DLG2* silenced, control and *DLG2* overexpressed THP1-ASC-GFP cells, in response to LPS and ATP treatment, as determined by PYCARD/ASC speck formation, presented as a percentage of observed cells. **g** The transfection efficiency of DLG2 in THP1 cells for silenced, control and DLG2 overexpression. Each experiment was performed in triplicate. The expression data are presented as log2 fold change and plotted as mean ± SD. *p < 0.05, **p < 0.01, ***p < 0.001

Using differentiated THP-1 monocytes we compared mock transfection to *DLG2* overexpressed cells and treated the cells with either growth medium, LPS or LPS with ATP. We determined the gene expression level of *IL1B*, *IL6*, *BAX* and *BCL2* (Fig. 4a–d) and NLRP3 (supplementary 2). *IL1B* showed higher expression in the *DLG2* transfected cells regardless of activation when compared to the equivalent activation (log2FC = 5.45, *p* < 0.001, log2FC = 3.22, *p* < 0.01 and log2FC = 3.03, *p* < 0.01, for the control, LPS and LPS + ATP respectively; Fig. 4a). *DLG2* attenuated *IL6* expression after activation with LPS and LPS and ATP (log2FC = 2.78, *p* < 0.001 and log2FC = 3.29, *p* < 0.001), with no difference in non-activated cells (Fig. 4b). *DLG2* overexpression also resulted in consistently higher *BAX* expression across all activations (log2FC = 1.45, *p* < 0.01, log2FC = 1.90, *p* < 0.001 and log2FC = 1.57, *p* < 0.01; Fig. 4c) and consistently lower *BCL2* expression across all activations (log2FC = 0.99, *p* < 0.01, log2FC = 1.01, *p* < 0.01 and log2FC = 1.33, *p* < 0.01; Fig. 4d). We subsequently determined the protein expression by immunoblot for DLG2, TIG1, BAX, BCL2, ser727 p-STAT3, total STAT3, ser235/236 p-S6, total S6 and GAPDH (Fig. 4e). *DLG2* overexpression resulted in increased BAX expression in non-activated, LPS and LPS + ATP stimulated cells, and protein expression of BCL2 was decreased across all activations, which agreed with the gene expression data. STAT3 phosphorylation increased stepwise in the mock transfected cells with the LPS + ATP treatment showing the highest phosphorylation. The overexpression of *DLG2* resulted in an increase of STAT3 phosphorylation during the LPS only treatment and a subsequent decrease during the LPS + ATP treatment (Fig. 4e). Furthermore, p-S6 was also decreased in all *DLG2* transfections while total S6 expression remained unaffected (Fig. 4e). The expression of TIG1 is increased in the control activations compared to the controls. As we previously determined overexpression of *DLG2* resulted in an increase in *IL1B* gene expression across all conditions. Finally, we investigated PYCARD/ASC speck formation in THP1 cells. We detected that *DLG2* overexpression resulted in an increase in PYCARD speck formation (8.3% more, *p* < 0.01; Fig. 4f) and *DLG2* silencing inhibited PYCARD speck formation (6.99% less, *p* < 0.01; Fig. 4f) in THP1 cells with stably transfected GFP tagged PYCARD compared to the control. To confirm the transfection efficiency, we determined DLG2 protein by immunoblot (Fig. 4g).

### DLG2 activated inflammasomes result in decreased colon cell proliferation

**Fig. 5:**
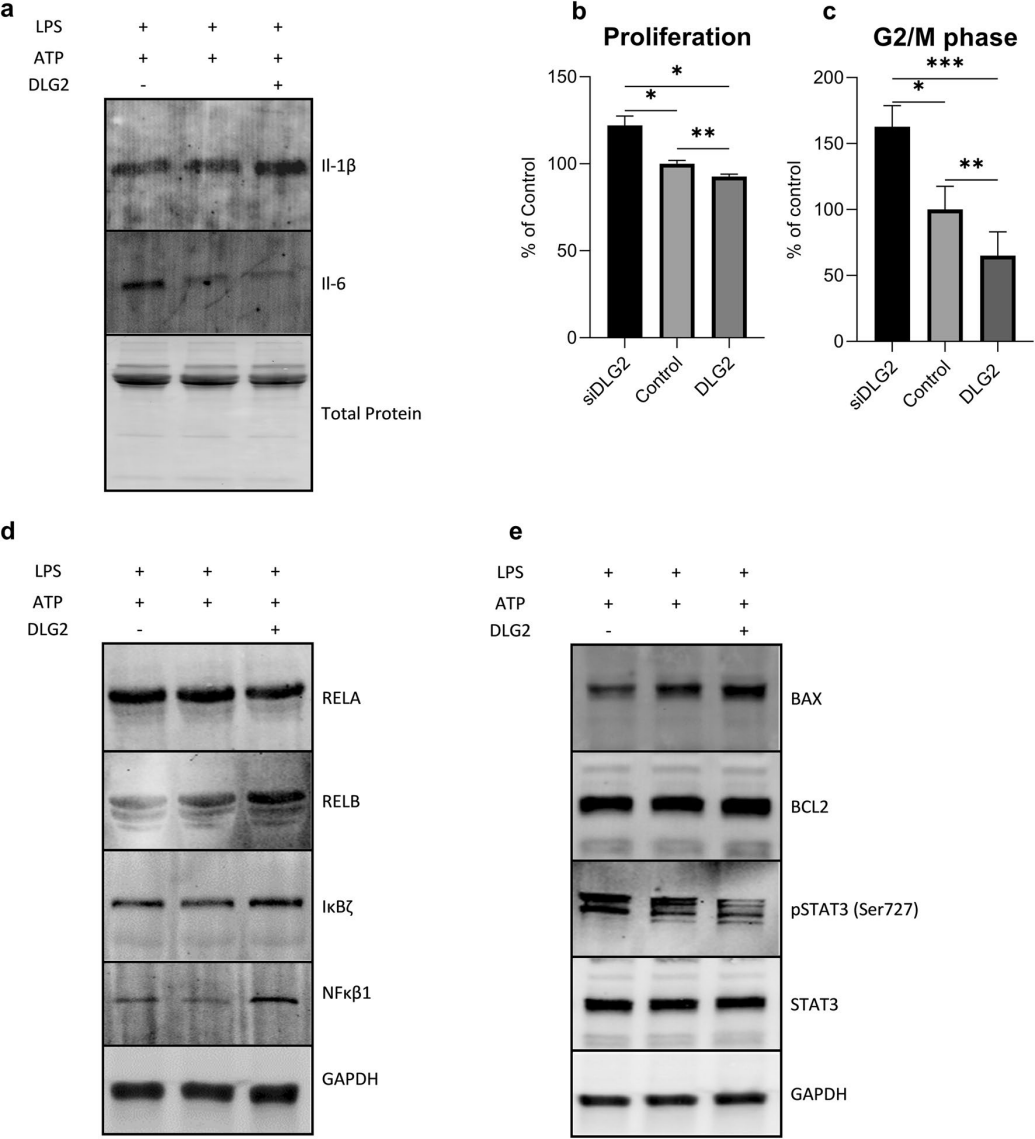
The response of THP-1 cytokine production in colon cancer COLO205 cells. **a** Representative western blot showing the level of IL-1β and IL-6 production and release in the cell medium in *DLG2* silenced, control and *DLG2* overexpressed THP-1 cells in response to LPS and ATP, normalized to total protein level. **b** The effect of cell medium from *DLG2* silenced (siDLG2), control and *DLG2* overexpressed (DLG2) THP-1 cells on COLO205 cell proliferation, and **c** percentage of COLO205 cells in G2/M phase. Representative immunoblot showing the effect of THP-1 inflammasome growth media on the expression of **d** RELA, RELB, IκBζ, NFκB1, **e** BAX, BCL2, p-STAT3, STAT3, normalized to GAPDH. Each experiment was performed in triplicate. The expression data are presented as log2 fold change and plotted as mean ± SD. *p < 0.05, **p < 0.01, ***p < 0.001

To model the effect of inflammasome formation on adjacent colon cancer cells we first quantified the amount of IL-1β and IL-6 in the supernatant taken from the transfected and activated THP-1 cells by immunoblot (Fig. 5a). We could show that silencing of *DLG2* expression resulted in a slight decrease of IL-1β and a strong increase in IL-6, while overexpression of *DLG2* had the opposite effect (Fig. 5a). We subsequently tested if the altered expression in IL-6 and IL-1β would affect the tumor microenvironment and modify signaling in colon cancer cells by treating COLO205 cells with the supernatant from THP1 transfected cells combined with regular growth media (1:1) followed by cell growth for 72 h. We detected that the *DLG2* knockdown THP1 cell media increased the proliferation of COLO205 (22.0% more cells/ml, *p* < 0.05; Fig. 5b) as well as increasing the proportion of cells in G2/M phase (62.8% more G2/M cells, *p* < 0.05; Fig. 5c) when compared to the control cells. *DLG2* overexpression resulted in the opposite of this, decreasing the cell proliferation (7.3% less cells, *p* < 0.01; Fig. 5b), and the number of cells in G2/M (34,9% less G2/M cells, *p* < 0.01; Fig. 5c). To show that NFκB and apoptosis signaling pathways were affected in response to these treatments we visualized protein expression of DLG2, RELA, RELB, IκBζ, NFKB1, BCL2, BAX, p-STAT3 Ser727, total STAT3 and GAPDH by immunoblot. These results showed that media from THP-1 *DLG2*-silenced cells decreased protein expression of RELB and BAX, and increased ser727 phosphorylation of STAT3 in COLO205 cells (Fig. 5d, e). Media from THP 1 *DLG2* overexpressed cells resulted in increased protein level of RELB, NFκB1 and decreased level of phosphorylation of STAT3 (Fig. 5d, e).

### *DLG2* expression was low in colon tumors and controlled signaling pathways

**Fig. 6:**
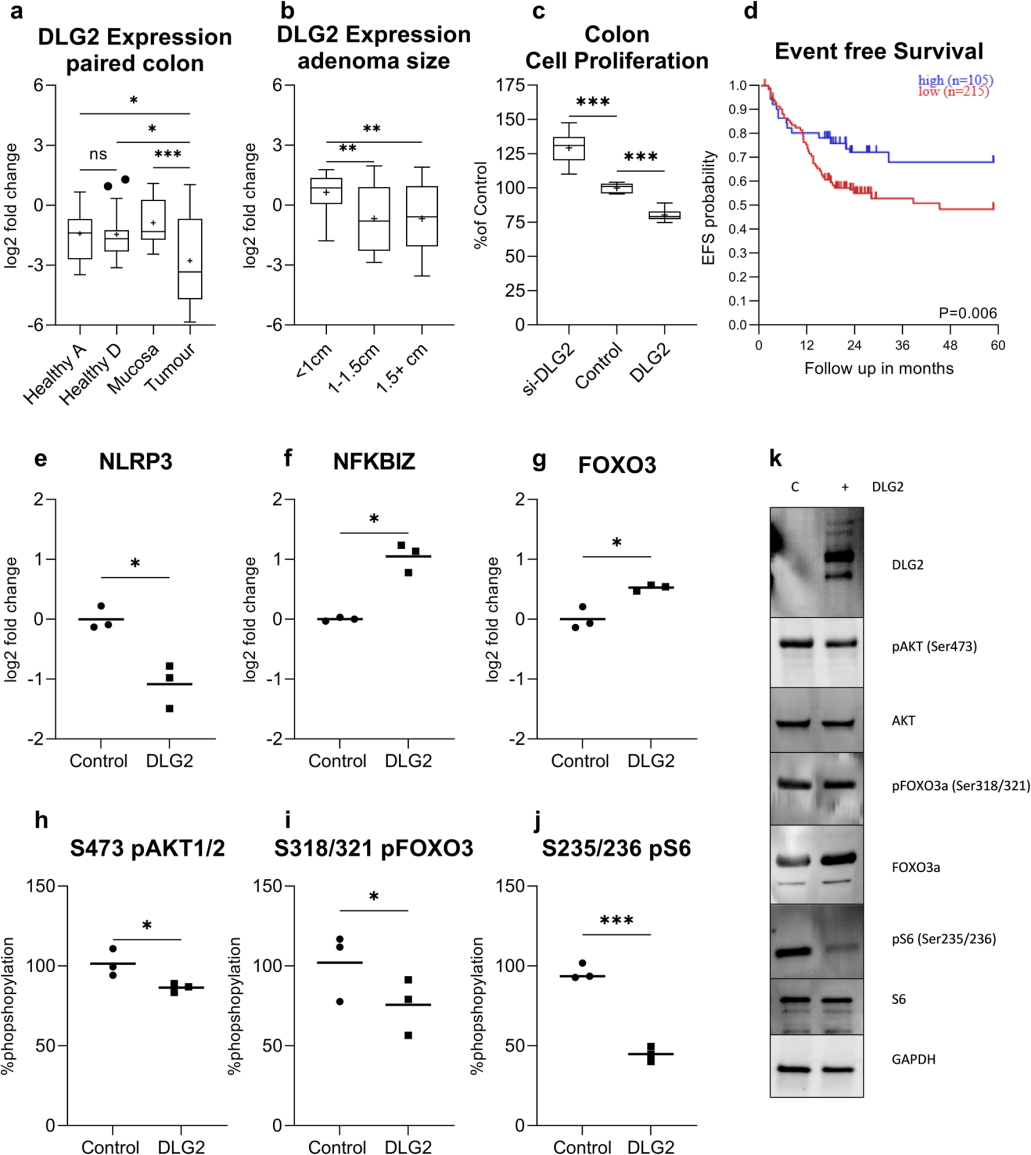
The expression of *DLG2* in colon cancer and the effect of low DLG2 level in colon cancer cells. **a** The expression of *DLG2* in 20 paired samples from the healthy ascending colon (Healthy A), healthy descending colon (Healthy D), mucosa from colon cancer patients 10 cm adjacent to the tumor (Mucosa) and colon cancer tumor (Tumor). **b** The*DLG2* expression in colon adenomas comparing 32 samples < 1 cm, 16 samples between 1 and 1.5 cm and 15 samples larger than 1.5 cm. **c** The effect of *DLG2* silencing (siDLG2) and overexpression (DLG2) on SW480 cell proliferation 48 h post transfection. **d** Kaplan–Meier survival curve showing the 5-year event free survival probability of colon cancer patients. **e–g** The gene expression of **e** *NLRP3*, **f** *NFKBIZ*, and **g** *FOXO3* in response to *DLG2* overexpression in SW480 cells. Normalized level of protein phosphorylation of **h** AKT1, **i** FOXO3 and **j** S6 in response to *DLG2* overexpression in SW480 cells. **k** Representative immunoblot showing the effect of *DLG2* overexpression on SW480 cells showing the expression of DLG2, p-AKT (s473), AKT1, p-FOXO3a (s318/321), FOXO3, p-S6 (s235/236) and S6, normalized to GAPDH. Each experiment was performed in triplicate. The expression data are presented as log2 fold change and plotted as either Tukey’s box and whisker plots showing IQR, line at the median, + at the mean with whiskers ± 1.5-fold of interquartile range or as mean. *p < 0.05, **p < 0.01, ***p < 0.001

We could show that *DLG2* gene expression was not significantly different between the ascending and descending colon in healthy controls (Log2 fold change = 0.04, *p* > 0.05; Fig. 6a). *DLG2* expression in the tumor tissue was lower than the paired mucosa sample (Log2 fold change = 1.89, *p* < 0.001), as well as the paired ascending and descending colon mucosa from the distal healthy colon tissue (Log2 fold change = 1.36, *p* < 0.05, Log2 fold change = 1.32, *p* < 0.05, respectively; Fig. 6a). Using publicly available microarray colon adenoma data (Sabates-Beliver et al. 2007) (GSE8671) we determined the expression of *DLG2* relative to adenoma size. We could also show that *DLG2* expression decreased as colon adenoma size increased to 1.1–1.5 cm and larger than 1.5 cm when compared to tumors under 1 cm in diameter (Log2 fold change = 1.32, *p* < 0.01 and Log2 fold change = 1.32, *p* < 0.01; Fig. 6b). To confirm these results, we determined proliferation in the colon cancer cells SW480 after *DLG2* silencing or overexpression, *DLG2* silencing resulted in an increase in SW480 proliferation (29.4% more cells, *p* < 0.001; Fig. 6c) and overexpression resulted in a decrease in proliferation (19.6% less cells, *p* < 0.001; Fig. 6c) compared to the control, 48 h after transfection. Using the dataset (Agesen et al. 2012) (GSE24551) to determine the if the expression of DLG2 resulted in altered survivability in colorectal cancer patients we performed a Kaplan–Meier survival analysis, with high DLG2 expression increasing the probability of 5-year patient survival.

We detected lower level of *NLRP3* (Log2 fold change = − 1.1, *p* < 0.05; Fig. 6e), and higher levels of *NFKBIZ* (Log2 fold change = 1.05, *p* < 0.05; Fig. 6f) and *FOXO3* (Log2 fold change = 0.53, *p* < 0.05; Fig. 6g) gene expressions after *DLG2* overexpression and compared to the mock transfection. At the protein level we subsequently determined that the percentage of phosphorylation of AKT (15.0%, *p* < 0.05; Fig. 6h), FOXO3 (26.4%, *p* < 0.05; Fig. 6i), and S6 (51.2%, *p* < 0.05; Fig. 6j) was lower in Sw480 cells overexpressing *DLG2*, as visualized in a representative immunoblot (Fig. 6k).

## Discussion

The digestive system is a complex series of organs that contains a high percentage of immune cells in order to protect the body from pathogens in the event of disruption of the physical mucosal and epithelial barrier. If the barrier is broken, invading pathogens trigger a PRR immune response and can induce inflammasome formation, the purpose of which is to induce an immune cascade to prepare the innate immune system as well as initiate repair of the physical barrier. Autoimmune diseases with chronic inflammation often separate these functions with chronic immune cascade signaling and minimal repair.

In this study we showed that *DLG2* was downregulated in human colon tumor tissue with the lowest *DLG2* level seen in larger size adenomas, and that silencing of *DLG2* caused an increase in colon cancer cell proliferation in vitro. Previously, it has been established that *DLG2* directly binds *FASL* which has been shown to mediate *NLRP3* inflammasome mediated apoptosis in liver metastases (Dupaul-Chicoine al. 2015). Here we show that overexpression of *DLG2* resulted in increased *NFKBIZ* expression, which is required for inflammasome activation (Horber et al. 2016). Increased expression of *NFKBIZ* is also known to curtail STAT3 activity and inhibit proliferation (Wu et al. 2009). Previous reports identified mutations in *NFKBIZ* in colon cancer which disrupt a stop codon, producing an abnormally long *C*-terminal region. This mutation is thought to affect the interactions with nuclear factor-κB complexes that bind to that region, altering the transcriptional regulation of its target genes and leading to cancer predisposition (Esteban-Jurado et al. 2015). Furthermore, *NFKBIZ* has been shown to be downregulated in bladder cancer and to affect the PI3K/AKT/mTOR pathway to inhibit proliferation (Xu et al. 2021). To test if *DLG2* influenced these pathways we investigated the phosphorylation levels of AKT, FOXO3 and S6 in colon cancer cells. We could show that *DLG2* overexpression reduced AKT phosphorylation at S473, the phosphorylation level of FOXO3 was maintained but the total protein was increased resulting in a decrease in the percentage of phosphorylation. The largest effect in response to *DLG2* overexpression was observed in the decrease in phosphorylated S6, highlighting that mTORC1 signaling seems to be altered with *DLG2* expression. High levels of phosphorylated S6 in colon cancer have been shown to be related to high nodal metastasis and high tumor histologic grade (Lai et al. 2014). Previous results have shown that the colon of colitis animal models have a high degree of phosphorylation of S6 suggesting that mTOR is involved in the disease, with inhibition of mTOR attenuating DSS induced colitis (Hu et al. 2016). In the ulcerative colitis (UC) patient data, in accordance with previous literature, *NFKBIZ* was upregulated in UC patients and even more in active UC cases, whereas we could show that *DLG2* was downregulated.

To investigate if *DLG2* downregulation is a direct result of inflammation, we investigated the effect of inflammation on *DLG2* gene expression in mouse, cell and fly models. We could show that acute inflammation initially resulted in an increase in *DLG2* expression across all models with a subsequent decrease over time, whereas *NLRP3* and *NFKBIZ* trended to increase over time. The initial increase and subsequent decrease in *DLG2* expression in response to inflammation implies that *DLG2* could respond to an inflammation feedback loop. To validate if *DLG2* expression affected the inflammatory cell response we investigated the formation of inflammasomes in THP1 cells after *DLG2* silencing, which resulted in decreased inflammasome formation after LPS and ATP stimulation (supplementary 3). Additionally, we were able to show that overexpression of *DLG2* resulted in increased *NFKBIZ*, *RELB*, *NFKB1* and IL-1β expression but a decrease in IL-6. It has previously been shown that *DLG2* increases p53 expression (Keane et al. 2022), which can inhibit IL-6 expression (Zhang et al. 2016) and cause IL-1β mediated cell cycle arrest (Guadagno et al. 2015). The homodimerization of p50 combined with IκBζ results in the transcription of anti-inflammatory genes and functions as a tumor suppressor (Cartwright et al. 2016). Previously, it has been shown that *NFKBIZ* prepares the priming stage of inflammasome formation by controlling *NLRP3* and pro IL-1β expression. The second signal required for inflammasome formation in the colon is the release of ADP by injured cells (Zhang et al. 2020a, b). Additionally, ATP is commonly thought of as an activator and extracellular levels of ATP are also commonly elevated in the tumor microenvironment (Di Virgilio and Adinolfi 2017; Alvarez et al. 2021). It has been noted that this signal results in an increase of IL-1β with marginal effect on TNF-α and IL-6. We show that p-P105 Ser932, which is activated by TNF-α, remains constant in the control activations and the DLG2 transfected activations, implying that TNF-α levels are also stable.

This work builds on the growing body of evidence that *DLG2* functions as a tumor suppressor. Recently, low *DLG2* expression has been found in osteosarcoma (Shao et al. 2019), ovarian cancer (Zhuang et al. 2019) and neuroblastoma (Keane et al. 2020, 2021; Siaw et al. 2020). The limitation of these studies has been that the mechanism and function of *DLG2* has not been directly shown, but generally inferred from bioinformatic analysis. Here we show that *DLG2* is downregulated in inflammatory bowel diseases such as UC as well as colon cancers, indicating that *DLG2* alteration occurs early in the tumorigenesis process. We also show that the downregulation is directly dependent on inflammation. The effect of *DLG2* loss is lowered expression of *NFKB1* and *NFKBIZ,* both of which are protective against carcinogens that cause genotoxic damage, providing a molecular mechanism for previous results showing that *DLG2* maintains genome integrity (Keane et al. 2022). Furthermore, previous bioinformatic results suggested that *DLG2* is involved in DNA replication (Keane et al. 2020), the cell cycle (Keane et al. 2020), apoptosis (Shao et al. 2019) and chemokine signaling (Shao et al. 2019). We have been able to confirm in colon cancer cells that increased *DLG2* results in an increase in *BAX* and a decrease in *BCL2* resulting in lower cell proliferation. Additionally, *DLG2* overexpression in THP-1 cells resulted in an altered cytokine and growth factor profile which was subsequently used to treat colon cancer cells decreasing the number in G2/M cell cycle phase. Which may be due to cell death caused by pyroptosis, however this would need to be further evaluated. Furthermore, a decrease in the phosphorylation of S6, consistent with control of proliferation was detected after *DLG2* overexpression. Decreased S6 phosphorylation has been shown to inhibit the synthesis of the chemokine IL-8 (Ang et al. 2019). Finally, *DLG2* loss has been shown to increase cyclin A2 and result in S phase progression and DNA replication (Keane et al. 2020), a similar function to the integral inflammasome component GSDMD (Wang et al. 2018).

Modulation of the immune system to treat cancers has been increasing over time, however, due to the complex nature and function of both the digestive system and immune system there has been some difficulty in developing immunotherapies for colon cancer. In this study we have shown that *DLG2* in macrophage like cells can activate the formation of the inflammasome. The suppression of *DLG2* seen in chronic inflammatory disease patients is one of the early changes that occurs and facilitates the formation of tumors. Restoration of *DLG2* in the colon may provide a mechanism for improved immunotherapy function as well as attenuating inflammatory bowel diseases. However, further work will be needed to evaluate the therapeutic potential of *DLG2* modulation.

## Supplementary Information

Below is the link to the electronic supplementary material.

The gene expression of (a) IL1N and TGFB1, and (b) IL4, IL10, IL13, IL27 or IL37 by sample type including; 8 Healthy patients, 15 IBD patients, 15 adenoma patients and 15 colon cancer patients (cohort GSE4183). IL1RN showing increased expression in IBD, adenoma and CRC compared to healthy controls. Data is plotted as mean ± SD. *p < 0.05, **p < 0.01, ***p < 0.001 (TIF 1939 KB)

The gene expression of NLRP3 under mock or DLG2 transfection combined with the three activation stages; basal, activation by LPS and activation by LPS + ATP. Each experiment was performed in triplicate. The gene expression data are presented as log2 fold change and plotted as mean ± SD (TIF 1129 KB)

Representative images of inflammasome formation as determined by PYCARD/ASC speck formation, observed in THP1 cells activated with LPS + ATP for; (a) DLG2 silenced, (b) Mock transfection or (c) DLG2 overexpression (TIF 3378 KB)

Supplementary file4 (DOCX 15 KB)

## Data Availability

The datasets generated during and/or analysed during the current study are available in the R2: genomics analysis visualization platform, http://r2.amc.n listed in the methods by GSE identification number.

## Abbreviations

CAC: Colitis associated colorectal cancer
SCC: Spontaneous colorectal cancer
TLR: Toll like receptors
PRR: Pattern recognition receptor
RIG-I: Retinoic acid inducible gene-I
EMT: Epithelial-mesenchymal transition
*RARRES1*: Retinoic acid response element 1
IBD: Inflammatory bowel disease
UC: Ulcerative colitis
DSS: Dextran sulfate sodium
