# Supplementary figures and images for: Inflammation suppresses DLG2 expression decreasing inflammasome formation
Source: J Cancer Res Clin Oncol. 2022 May 2;148(9):2295–311. doi: 10.1007/s00432-022-04029-7 (PMC9349146; doi:10.1007/s00432-022-04029-7)

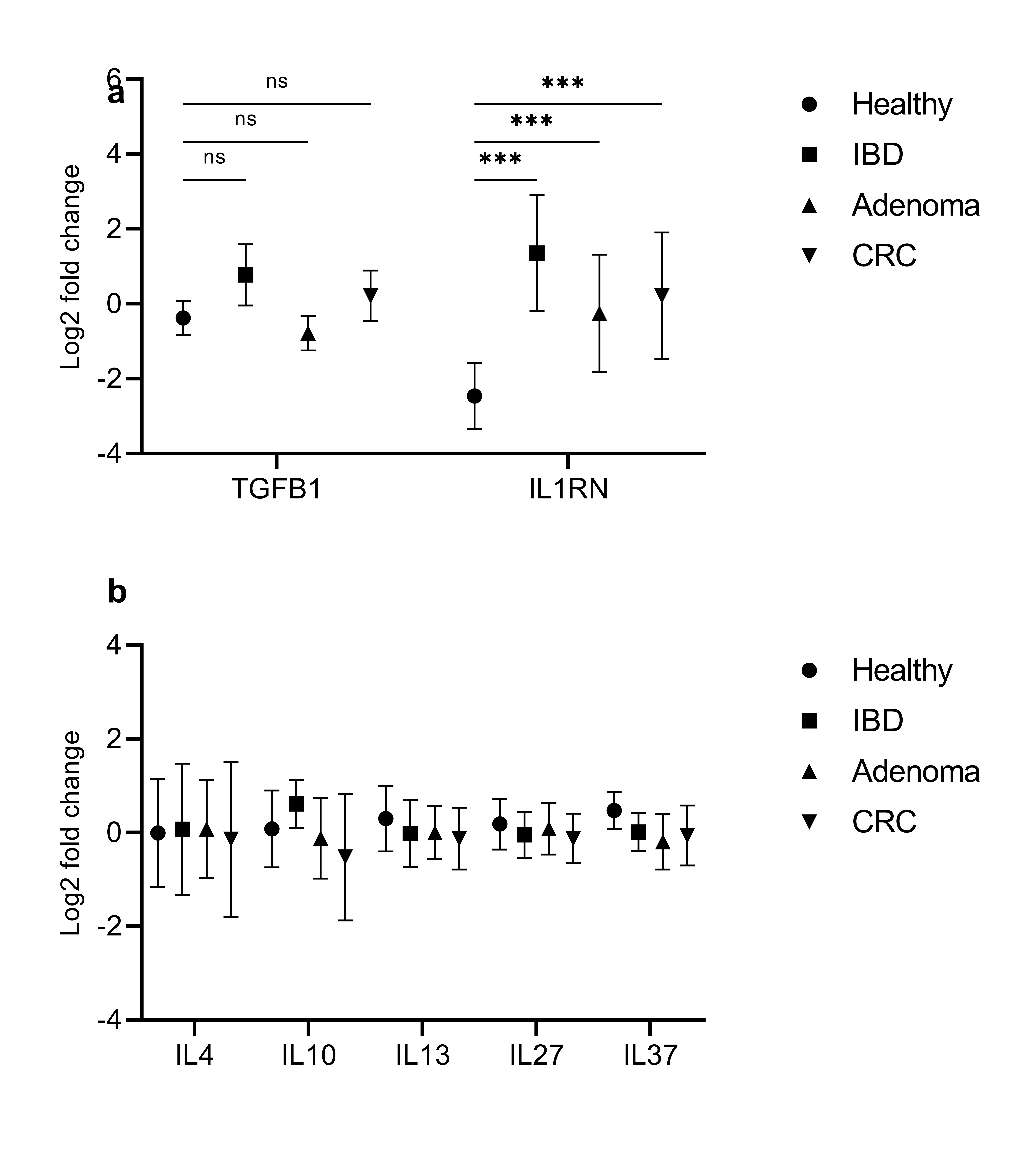

Supplement: Supplementary file 1 — The gene expression of (a) IL1N and TGFB1, and (b) IL4, IL10, IL13, IL27 or IL37 by sample type including; 8 Healthy patients, 15 IBD patients, 15 adenoma patients and 15 colon cancer patients (cohort GSE4183). IL1RN showing increased expression in IBD, adenoma and CRC compared to healthy controls. Data is plotted as mean ± SD. *p < 0.05, **p < 0.01, ***p < 0.001 (TIF 1939 KB) [file 432_2022_4029_MOESM1_ESM.tif]

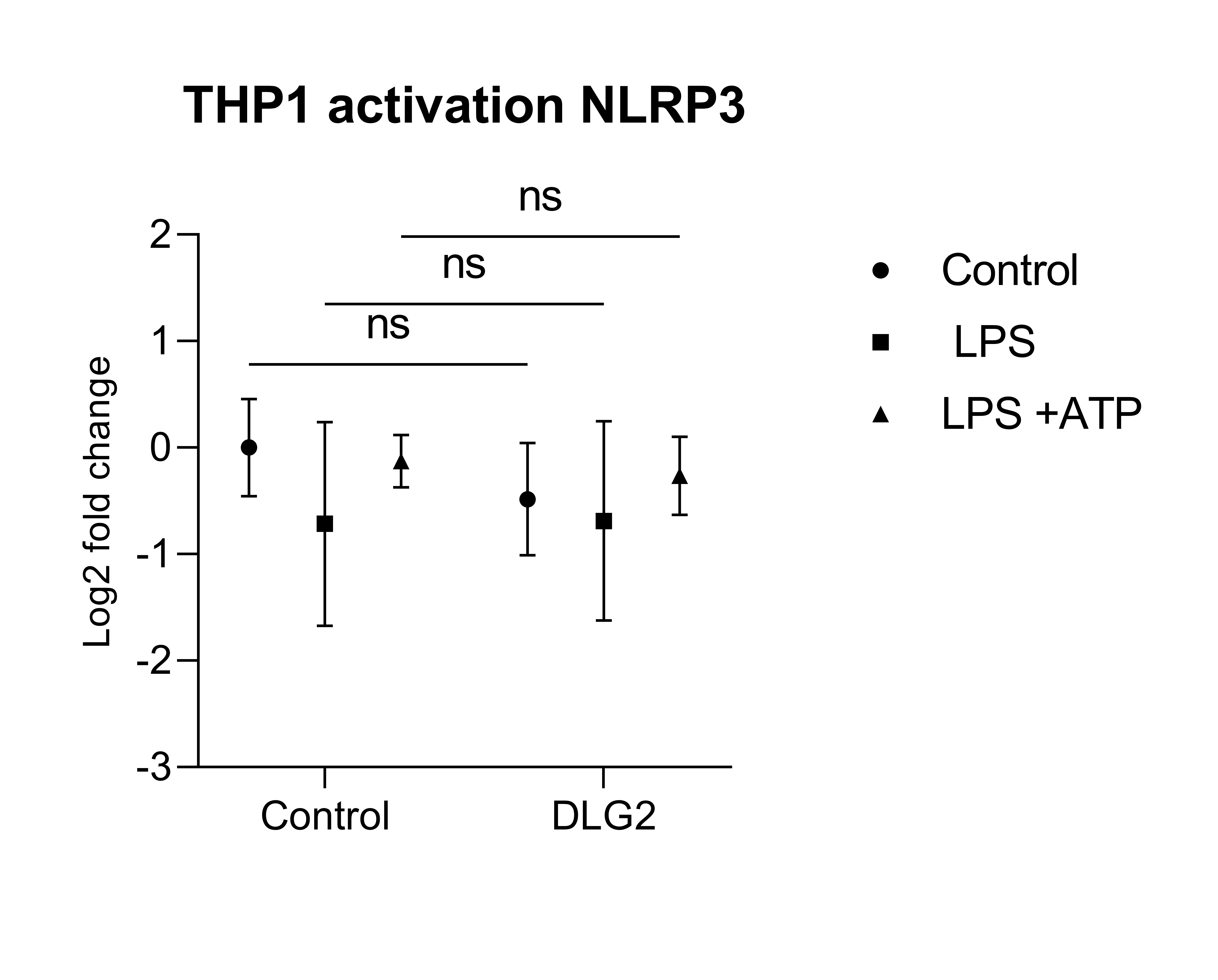

Supplement: Supplementary file 2 — The gene expression of NLRP3 under mock or DLG2 transfection combined with the three activation stages; basal, activation by LPS and activation by LPS + ATP. Each experiment was performed in triplicate. The gene expression data are presented as log2 fold change and plotted as mean ± SD (TIF 1129 KB) [file 432_2022_4029_MOESM2_ESM.tif]

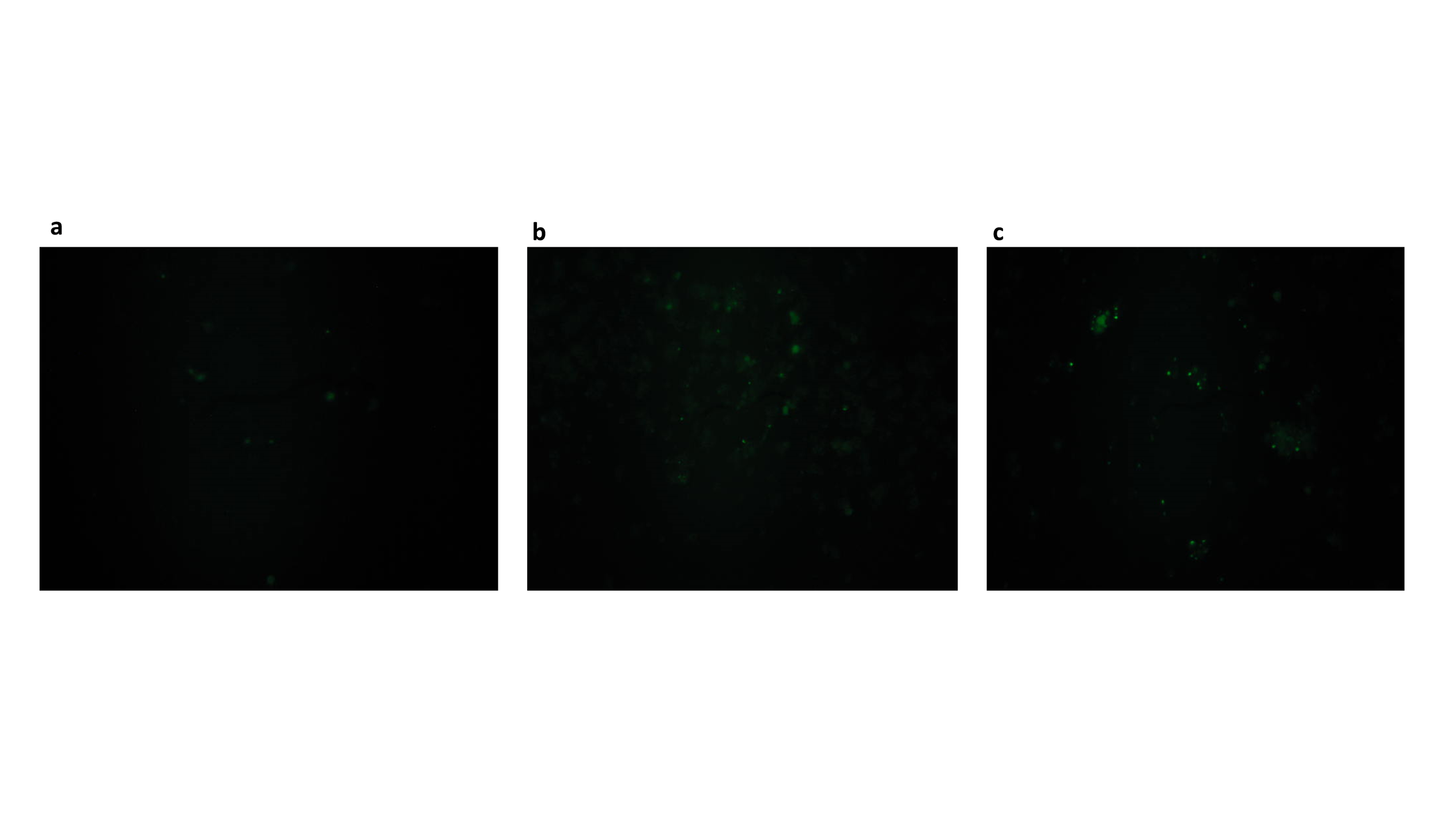

Supplement: Supplementary file 3 — Representative images of inflammasome formation as determined by PYCARD/ASC speck formation, observed in THP1 cells activated with LPS + ATP for; (a) DLG2 silenced, (b) Mock transfection or (c) DLG2 overexpression (TIF 3378 KB) [file 432_2022_4029_MOESM3_ESM.tif]
