# Supplementary material for: Inflammation suppresses DLG2 expression decreasing inflammasome formation
Source: J Cancer Res Clin Oncol. 2022 May 2;148(9):2295–311. doi: 10.1007/s00432-022-04029-7 (PMC9349146; doi:10.1007/s00432-022-04029-7)
Supplement: Supplementary file 4 — Supplementary file4 (DOCX 15 KB) [file 432_2022_4029_MOESM4_ESM.docx]

## Supplementary

| **Additional file 1.** Reason for referral of study participants to colonoscopy | |
| --- | --- |
| **Case** | **Reason referral** |
| 1 | Fatigue, low Hb, positive F-Hb |
| 2 | Iron deficiency, anemia |
| 3 | Rectal bleeding, low Hb |
| 4 | Anemia |
| 5 | Abdominal pain |
| 6 | Changed stool habits, rectal bleeding |
| 7 | Anemia |
| 8 | Abdominal pain |
| 9 | Rectal bleeding, low Hb |
| 10 | Iron deficiency, anemia |
| 11 | Rectal bleeding |
| 12 | Abdominal pain |
| 13 | Follow-up after adenoma |
| 14 | Anemia, rectal bleeding |
| 15 | Rectal bleeding |
| 16 | Changed stool habits |
| 17 | Fatigue, anemia |
| 18 | Rectal bleeding, changed stool habits |
| 19 | Anemia |
| 20 | Changed stool habits, positive F-Hb |
